# Prevalence and clinical profile of abnormal glucose in first-episode and drug-naïve patients with major depressive disorder with comorbid abnormal thyroid function: a large-scale cross-sectional study

**DOI:** 10.1186/s12888-023-04842-5

**Published:** 2023-05-24

**Authors:** Xiao Huang, Yuan Sun, Anshi Wu, Xiang-Yang Zhang

**Affiliations:** 1 Department of Anesthesiology, Beijing Chao-Yang Hospital, Capital Medical University, No. 8 Workers’ Stadium South Road, Beijing, Chaoyang 100020 China; 2 Department of Pharmacy, Beijing Chao-Yang Hospital, Capital Medical University, No. 8 Workers’ Stadium South Road, Beijing, Chaoyang 100020 China; 3 CAS Key Laboratory of Mental Health, Institute of Psychology, Beijing, China; 4 Department of Psychology, University of Chinese Academy of Sciences, Beijing, China; 5 Key Laboratory of Mental Health, Institute of Psychology, Chinese Academy of Sciences, 16 Lincui Rd, Beijing, 100101 China

**Keywords:** Depression, Glucose metabolism, Thyroid function, Prevalence

## Abstract

**Background:**

The associated factors of abnormal glucose in patients with major depressive disorder (MDD) with comorbid abnormal thyroid function (ATF) remain unclear. To the best of our knowledge, this is the first study with a large sample size that examines the risk factors of abnormal glucose in first-episode drug-naïve (FEDN) MDD patients comorbid with ATF and includes clinical correlates and thyroid hormone levels.

**Methods:**

A total of 1718 FEDN MDD patients were recruited. The Hamilton Depression Scale (HAMD), Hamilton Anxiety Scale (HAMA), and Positive and Negative Syndrome Scale (PANSS) positive subscale were used to evaluate the symptoms of patients. Fasting blood glucose concentration and thyroid hormone levels were measured.

**Results:**

The prevalence of abnormal glucose in MDD patients with comorbid ATF was 47.3%, which was 4.25 times higher than that in MDD patients without ATF (17.4%). Compared to those ATF patients without abnormal glucose, ATF patients with abnormal glucose scored higher on HAMD, HAMA and PANSS positive subscale, had a higher rate of suicide attempts, severe anxiety and psychotic symptoms, and had higher levels of thyroid-stimulating hormone (TSH), and thyroid peroxidases antibody (TPOAb) which were also correlated with abnormal glucose in MDD patients comorbid ATF (all *P* < 0.05). The combination of HAMD score and TSH could differentiate abnormal glucose from ATF. Further, TSH was independence-related with the concentration of fasting blood glucose in MDD patients with comorbid ATF.

**Conclusion:**

Our results demonstrate a high prevalence of abnormal glucose in MDD patients with comorbid ATF. Some clinical and thyroid function-related variables may be associated with abnormal glucose in MDD patients with comorbid ATF.

## Background

Major depressive disorder (MDD) is a common disorder among psychiatric patients characterized by significant mood changes and symptoms, including lack of enjoyment, low self-esteem, sleep disturbances, and cognitive changes [1, 2]. It is estimated to be the second most socioeconomically burdensome disease by 2020, affecting 10% of the global population [3, 4]. The 12-month and lifetime prevalence of MDD in adults is 10.4% and 20.6%, respectively, with the majority of lifetime MDD cases being moderate (39.7%) or severe (49.5%) [5].

Abnormalities in thyroid function are a common coexisting condition associated with MDD. Many studies have found a link between thyroid function abnormalities and mood disorders [6, 7]. Our recent study showed that the incidence of severe subclinical hypothyroidism (SCH) was 3.8% in MDD patients, and 4.7% in MDD patients over 40 years of age [8]. In addition, the prevalence of hypothyroidism is higher in patients with early-onset affective disorders than in those with late-onset affective disorders [9].

Many studies have also confirmed the complex relationship between glucose metabolism disorders and mood disorders. The prevalence of depression is higher in patients with type 1 or type 2 diabetes than in the general population and is associated with a poor prognosis [10–12]. A systematic analysis by Roy et al. showed that patients with diabetes are at increased risk for depression [13]. Altered glucose metabolism is one of the pathways implicated in the pathophysiology of MDD [14]. There is growing evidence that suggests that depression and type 2 diabetes share a common biological origin, possibly through dysregulation of the hypothalamic-pituitary-adrenal axis [15]. Depressive symptoms are closely associated with severe hypoglycemia and diabetic ketoacidosis. Successful treatment of depressive symptoms may lead to improved long-term outcomes in diabetes [16].

Many studies have found a complex association between thyroid dysfunction and abnormal glucose. For example, Liu et al. showed that changes in free triiodothyronine (FT3) and total FT3 levels were positively associated with changes in body weight, body fat percentage, glucose and insulin over 6 and 24 months. Higher baseline free FT3 and free thyroxine (FT4) predicted more weight loss, but no weight gain in overweight and obese adults with normal thyroid function [17]. Mullur et al. also found that thyroid dysfunction resulted in significant changes in body weight and resting metabolic rate (RMR) [18]. Thyroid hormones play an important role in energy regulation. In normal thyroid function, non-diabetic, obese adolescent males, there is a sex-specific association between thyroid-stimulating hormone (TSH) and insulin sensitivity [19]. According to Weiss et al., many overweight and obese adolescents exhibit a metabolic syndrome characterized by insulin resistance and dyslipidemia [20]. Tight glucose control (TGC) further exacerbates peripheral inactivation of thyroid hormones [21]. Although many studies have highlighted the possible association between abnormal thyroid function (ATF) and depression, few studies have investigated the relationship between ATF and abnormal glucose in patients with MDD. Therefore, whether ATF is a risk factor for abnormal glucose in MDD patients remains uncertain.

To our knowledge, this was the first study to explore the prevalence and clinical profile of abnormal glucose in first-episode and drug-naïve (FEDN) MDD patients with comorbid ATF. We aimed (1) to examine the prevalence and clinical profile of abnormal glucose in FEDN MDD patients with comorbid ATF and (2) to identify predictors significantly associated with abnormal glucose in FEDN MDD patients with comorbid ATF.

## Materials and methods

### Subjects

A total of 1718 patients were included in this cross-sectional study between 2015 and 2017. Inclusion criteria included: (1) being between 18 and 60 years of age and Han Chinese; (2) meeting the diagnostic criteria for MDD, according to the Diagnostic Interview of the Structured Clinical Interview for DSM-IV (SCID), which was conducted by two experienced clinical psychiatrists; (3) not previously treated with antidepressants or any other specific medications; and (4) having a 17-item Hamilton Rating Scale for Depression (HAMD) score ≥ 24 and current depressive symptoms were the first episode. Exclusion criteria included: (1) severe or unstable physical illness; (2) meeting any other major Axis I disorder; (3) drug or alcohol abuse or dependence, except for nicotine; (3) refusal to sign a written consent form; (4) patients with the history of drug use that influenced the blood glucose level. For the purpose of this study, we defined the first episode as the first symptomatic episode.

This study was conducted in accordance with the Declaration of Helsinki and was approved by the Institutional Review Boards of First Hospital of Shanxi Medical University (ID number: 2016-Y27). Written informed consent was obtained from all participants.

### Data collection and assessment

Sociodemographic and clinical data including age, sex, body mass index (BMI), age of onset, duration of illness, education level, and marital status were collected directly from each patient. Additional information was collected from medical records and other data sources.

The severity of depressive or anxiety symptoms in all participants was assessed using the 17-item HAMD and the 14-item Hamilton Anxiety Scale (HAMA), respectively. The HAMD-17 consists of 17 items, 8 items of which are scored on a 5-point scale from 0 (not present) to 4 (severe), and the remaining 9 items are scored from 0 (absent) to 2 (symptom-specific severity descriptor). In the present study, patients with a score of 24 or more were defined as having severe depressive symptoms [22, 23]. The HAMA consisted of 14 items, all of which are rated on a 5-point Likert scale (0: not present; 4: severe). In this study, participants were classified as having or not having severe anxiety symptoms with a cutoff point of 25 [24].

Psychotic symptoms were assessed by the Positive and Negative Syndrome Scale (PANSS) positive symptom subscale. Each item has a score from 1 to 7. In this study, patients who scored 15 or more on the PANSS positive symptom subscale were identified as having psychotic symptoms [25].

To ensure the reliability and consistency of the scales throughout the study, two trained clinicians performed the scale assessments. These two clinicians were unaware of the patients’ clinical status. The interrater correlation coefficients of two psychiatrists for the HAMD, HAMA, and PANSS scores were all greater than 0.8.

### Measurement of biochemical parameters

Fasting blood samples were collected from all patients at 6–8 am. Samples were measured by 11:00 am the same day. Serum levels of FT3, FT4, anti-thyroglobulin (TgAb), thyroid peroxide antibody (TPOAb), TSH and plasma glucose were measured using the Cobas E610 (Roche, Basel, Switzerland) chemiluminescent immunoassay. According to the American Diabetes Association, the threshold for abnormal glucose was 5.6 mmol/l of fasting plasma glucose [26]. ATF was defined as TSH > 4.2 mIU/L, with several previous studies using the same definition for ATF [27, 28].

### Statistical analysis

Data from all participants were anonymized according to the Declaration of Helsinki. The distribution of continuous variables was examined with the Kolmogorov-Smirnov one-sample test. Analysis of variance (ANOVA) was used for continuous data with normal distribution. Abnormally distributed continuous data were compared using the Mann-Whitney U test. The Chi-square test was used for categorical data between the two groups. Bonferroni correction was used to adjust for multiple testing. To investigate risk factors for abnormal glucose in FEDN MDD patients with comorbid ATF, ANOVA was performed on those with and without abnormal glucose. Significantly different factors were then included in logistic regression. The area under the receiver operating characteristic (AUC-ROC) was used to determine the discriminatory ability of significant parameters to distinguish patients with abnormal glucose from patients without abnormal glucose. In addition, multivariate regression analysis was performed to detect the relationship between fasting blood glucose concentration and clinical and biochemical correlates of MDD patients with comorbid ATF.

All data were analyzed using Social Sciences for Windows (SPSS) (version 25.0) (IBM, Chicago, IL, USA). Graphs were then plotted using GraphPad Prism 6.0. Coefficient values, odds ratios (OR), and 95% confidence intervals (CI) were used to quantify the strength of association. Two-tailed P values < 0.05 were considered statistically significant.

## Results

### Prevalence and clinical characteristics of abnormal glucose in MDD patients with and without Comorbid ATF

A total of 1718 eligible participants (M/F = 588/1130; age range 18–60 years) were enrolled in this study. The proportion of MDD patients with ATF was 60.8% (1044/1718). Compared with MDD patients without ATF, MDD patients with ATF were older, had a greater BMI, higher rates of suicide attempts, severe anxiety and psychotic symptoms, and higher scores on the HAMA, HAMD, and PANSS positive subscale (all *P* < 0.05, all *P* Bonferroni correction < 0.05). The prevalence of abnormal glucose was higher in MDD patients with ATF (n = 494, 47.3%) than in MDD patients without ATF (n = 117, 17.4%) (χ^2^ = 160.42, *P* < 0.001, OR = 4.28, 95% CI: 3.39–5.40). After controlling for age, gender, suicide, severe anxiety, psychotic symptoms, HAMD, HAMA, PANSS positive subscale, and BMI, the rate of abnormal glucose was 3.55 times higher in MDD patients with ATF than in MDD patients without ATF (B = 1.27, Wald statistic = 95.58, *P* < 0.0001, OR = 4.25, 95% CI = 2.76–4.58). (Table 1).

**Table 1:** Demographic and clinical characteristics of ATF in MDD patients

Variable	ATF (n=1044)	NTF (n=674)	F, Z or χ^2^	*P*
**Male, n (%)**	358(34.3)	230(34.1)	0.005	0.985
**Education, n (%)**			3.387	0.336
**1**	263(25.2)	150(22.3)		
**2**	451(43.2)	309(45.9)		
**3**	267(25.6)	182(27.0)		
**4**	63(6.0)	33(4.9)		
**Married, n (%)**	760(72.8)	456(67.7)	4.99	0.026
**Suicide, n (%)**	263(25.2)	83(12.3)	41.43	< 0.001
**Severe anxiety, n (%)**	160(15.3)	44(6.5)	29.46	< 0.001
**Exhibiting psychotic symptoms, n (%)**	136(13.0)	35(5.2)	27.18	< 0.001
**Abnormal glucose, n (%)**	494(47.3)	117(17.4)	159.11	< 0.001
**Duration of disease, month**	7.00(4.85)	5.23(4.33)	59.06	< 0.001
**Actual age, year**	35.68(12.42)	33.61(12.34)	11.44	0.001
**Actual age group**			14.4	0.001
**18–39, n (%)**	605 (58)	451 (66.9)		
**40–49, n (%)**	267 (25.6)	129 (19.1)		
**≥ 50, n (%)**	172 (16.5)	94 (13.9)		
**Age of onset, year**	35.44(12.28)	33.45(12.28)	10.72	0.001
**HAMD**	31.23(2.72)	28.86(2.69)	313.74	< 0.001
**HAMA**	21.18(3.64)	20.21(3.12)	32.40	< 0.001
**PANSS**	9.47(4.94)	7.89(3.15)	54.13	< 0.001
**Fasting blood glucose (mmol/L)**	5.60(0.63)	5.09(0.55)	291.60	< 0.001
**Fasting blood glucose group**			161.08	< 0.001
**< 5.6 mmol/L, n (%)**	550 (52.7)	557 (82.6)		
**5.6–6.9 mmol/L, n (%)**	471 (45.1)	114 (16.9)		
**≥ 7 mmol/L, n (%)**	23 (2.2)	3 (0.4)		
**BMI, kg/m** ^**2**^	24.62(1.99)	23.97(1.75)	48.08	< 0.001

### Clinical characteristics and biochemical parameters of MDD with Comorbid ATF with and without abnormal glucose

As shown in Table 2, ANOVA revealed significant differences in demographic and clinical characteristics between patients with and without abnormal glucose, including suicide attempts, severe anxiety, psychotic symptoms, HAMD score, HAMA score, and PANSS positive subscale score (all *P* < 0.05). Patients with abnormal glucose had higher levels of TSH and TPOAb compared to patients without abnormal glucose (all *P* < 0.001, all Bonferroni corrected *P* ≤ 0.001). Different age subgroups (18–39, 40–49, ≥ 50 years) analysis showed no significant difference between patients with and without abnormal glucose (χ^2^ = 0.75, P = 0.69).

**Table 2:** Socio-demographics and clinical characteristics between MDD comorbid ATF with and without abnormal glucose

Variable	MDD comorbid ATF		F, Z or χ^2^	*P*
	With abnormal glucose (n = 494)	Without abnormal glucose (n = 550)		
**Male, n (%)**	158(32.0)	200(36.4)	2.026	0.137
**Education, n (%)**			1.663	0.645
**1**	131(26.5)	132(24.0)		
**2**	213(43.1)	238(43.3)		
**3**	124(25.1)	143(26.0)		
**4**	26(5.3)	37(6.7)		
**Marry status, n (%)**	355(71.9)	405(73.6)	0.33	0.566
**Suicide, n (%)**	156(31.6)	107(19.5)	19.66	< 0.001
**Severe anxiety, n (%)**	90(18.2)	70(12.7)	5.63	0.018
**Exhibiting psychotic symptoms, n (%)**	81(16.4)	55(10.0)	8.84	0.003
**Actual age, year**	35.76(12.57)	35.61(12.30)	0.33	0.855
**Duration of disease, month**	7.06(4.57)	6.95(5.09)	0.13	0.715
**Age of onset, year**	35.51(12.43)	35.38(12.16)	0.03	0.862
**Actual age group**			0.75	0.69
**18–39 years, n (%)**	280 (56.7)	325 (59.1)		
**40–49 years, n (%)**	132 (26.7)	135 (24.5)		
**≥ 50 years, n (%)**	82 (16.6)	90 (16.4)		
**HAMD**	31.65(2.62)	30.85(2.74)	22.88	< 0.001
**HAMA**	21.66(3.70)	20.74(3.52)	16.77	< 0.001
**PANSS**	10.02(5.45)	8.97(4.39)	11.72	0.001
**TSH, mIU/L**	7.18(1.95)	6.23(1.65)	72.33	< 0.001
**A-TG, IU/Ml**	23.1(15.9,115.4)	22.5(14.9,60.2)	-1.18	0.237
**A-TPO, IU/Ml**	22.3(12.9,94.9)	19.7(12.4,44.7)	-3.26	0.001
**FT3, pg/mL**	4.91(0.73)	4.93(0.75)	0.24	0.625
**FT4, ng/dL**	16.64(3.09)	16.67(3.13)	0.02	0.885
**BMI, kg/m** ^**2**^	24.58(1.99)	24.66(1.94)	0.44	0.507

### Risk factors for abnormal glucose in ATF in patients with MDD

We then focused on the risk factors for abnormal glucose in MDD patients with ATF. Significantly different variables from the univariate analysis were included in logistic regression to detect risk factors for abnormal glucose in MDD patients with ATF. As shown in Table 3, the risk factors for abnormal glucose of ATF in MDD patients with ATF were as follows: HAMD score (B = 0.09, *P* = 0.01, OR = 1.10), and TSH levels (B = 0.28, *P* < 0.001, OR = 1.32). Moreover, age was not a risk factor for abnormal glucose in MDD with ATF (OR = 1, 95%CI = 0.99–1.02, *P* = 0.8). In addition, the AUCROC showed the following values for each risk factor: 0.589 for HAMD and 0.653 for TSH. We then combined these parameters and found an AUC value of 0.654 for the combination of HAMA score and TSH to distinguish between patients with abnormal glucose from patients without abnormal glucose.

**Table 3:** The risk factors of abnormal glucose in MDD patients with ATF

	B	Wald statistics	df	Sig.	OR	95%CI lower	95%CI upper
**Age**	0	0.06	1	0.80	1	0.99	1.02
**Duration of disease**	0	0.08	1	0.78	1	0.98	1.03
**Married**	-0.16	0.60	1	0.44	0.85	0.57	1.27
**Suicide**	0.17	1.01	1	0.32	1.19	0.85	1.66
**Severe anxiety**	-0.14	0.22	1	0.64	0.87	0.5	1.53
**Exhibiting psychotic symptoms**	0.46	0.48	1	0.49	1.59	0.43	5.84
**HAMD**	0.09	7.10	1	0.01	1.1	1.03	1.18
**HAMA**	-0.03	1.06	1	0.30	0.97	0.91	1.03
**PANSS**	-0.04	0.73	1	0.39	0.96	0.87	1.06
**TSH**	0.28	39.78	1	0	1.32	1.21	1.44
**A-TPO**	0	1.6	1	0.21	1	1	1

### Correlation of fasting blood glucose level with thyroid hormones parameters in MDD patients with comorbid ATF

**Fig. 1:**
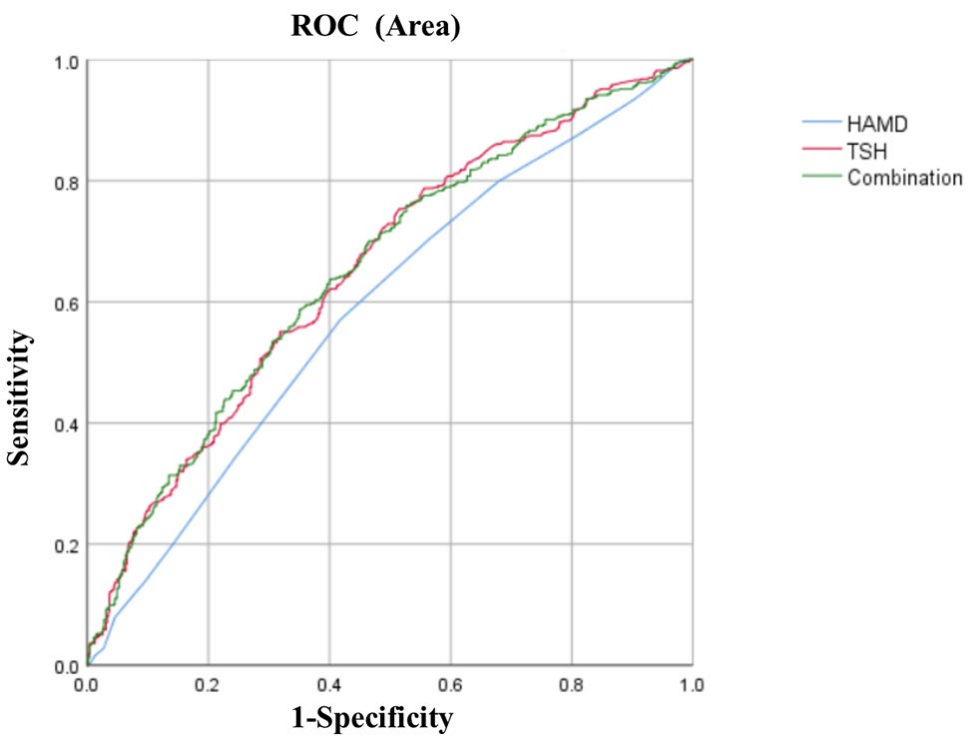
The discriminatory capacity of related factors for distinguishing between patients with and without abnormal glucose in MDD comorbid with ATF. The area under the curve of HAMD score, TSH, and the combination of these three factors were 0.589, 0.653, and 0.654, respectively. ROC: receiver operating characteristic. HAMD: Hamilton Rating Scale for Depression. TSH: thyroid stimulating hormone

Multivariate regression analysis showed that TSH levels (B = 0.11, t = 8.68, *P* < 0.001) were independently correlated with fasting blood glucose levels (Fig. 1).

## Discussion

To our knowledge, the present study is the first to examine the incidence and clinical characteristics of abnormal glucose in FEDN MDD with comorbid ATF. The main results of our study were as follows: (1) the prevalence of abnormal glucose in Chinese FEDN MDD patients with comorbid ATF was 47.3%; (2) higher HAMD scores and TSH levels were associated with abnormal glucose in FEDN MDD patients. Our study showed a high prevalence of abnormal glucose in Chinese FEDN MDD patients with comorbid ATF, and higher HAMD scores and TSH levels may contribute to abnormal glucose in MDD patients with ATF.

In the present study, the incidence of comorbid with abnormal glucose in FEDN patients with MDD and ATF was 47.3%. Additionally, the prevalence of abnormal glucose in MDD patients with comorbid ATF in this study was significantly higher than in those without ATF. Although several studies have reported the complex relationship between impaired blood glucose and ATF, as well as impaired blood glucose and mood disorders, no study has explored abnormal glucose in FEDN patients with MDD and ATF. The risk of abnormal glucose in MDD increased by 33% compared with the general population [29]. Karen et al. reported that the prevalence of abnormal glucose tolerance was14.6% in minor depression and 15.3% in major depression during pregnancy among Hispanic women respectively [30]. Hu et al. found that the rate of impaired Fasting Glucose in adolescent-onset patients with first-episode drug-naive schizophrenia was 21.6% [31]. Fugger et al. showed that the prevalence rate for comorbid diabetes among MDD patients was 6% [32]. All these differences in the prevalence may be due to the following reasons: First, the abnormal glucose defined in our analysis was fasting glucose greater than or equal to 5.6 and ATF was defined as TSH higher than 4.2 mIU/L, with the relatively lower thresholds compared with traditional criteria such as the oral glucose tolerance test (OGTT). We have to admit that these findings might be over-estimated. Second, the severity of MDD progression and the presence of multiple factors, as well as thyroid function, may affect blood glucose levels. In terms of endocrine function, the effects of TSH on glucose metabolism may vary depending on the pathway involved. Third, some mental illness and antipsychotics, may affect blood glucose concentrations [33, 34]. We have not ruled out all those confounding factors. Therefore our results therefore need to be interpreted with caution.

We showed that there was no significant difference in social demographic characteristics, including gender, age, age of onset, time of onset, level of formal education and marital status, between the normal and abnormal glucose in FEDN patients with MDD comorbid ATF groups. But comorbid abnormal glucose in MDD patients with ATF had a higher suicide attempt, anxiety and psychiatric symptoms rate, and higher HAMA score, HAMD score and PANSS score. Our results are consistent with the previous studies. Whitworth et al. have shown that lifelong depression and anxiety increased the risk of more severe psychological symptoms and hyperglycemia in patients with type 2 diabetes [35]. The combined prevalence of depressive symptoms among adolescents with type 1 diabetes was 30.04%, and 32% of patients reported symptoms of anxiety [11]. The systematic evaluation by OuYang et al. also revealed that anxiety or depression during pregnancy increased the incidence of gestational diabetes mellitus in pregnant women to some extent. Also, the diagnosis of gestational diabetes mellitus increases the rate of anxiety and depression in pregnant women [36]. Treatment of gestational diabetes reduced the incidence of severe perinatal depression and also improved woman’s health-related quality of life [37]. We hypothesize the reason patients with comorbid abnormal glucose in MDD patients with ATF showed a higher suicide attempt, anxiety and psychiatric symptoms rate is that the biological link between abnormal glucose and MDD may be interfered with by these factors. The complex interaction between abnormal glucose and MDD may be confounded by others and required to be further explored in the future.

Multiple factors have been reported to affect glucose metabolic function in patients with MDD. In the present study, we found that in MDD patients with comorbid ATF, clinical features and biochemical parameters were associated with abnormal glucose, including HAMD score and TSH. Previous studies have consistently shown that the severity of depression and TSH in MDD patients increased abnormal glucose. Many previous studies have suggested that thyroid metabolic parameters and HAMD may be risk factors for mood disorders. For instance, the randomized, placebo-controlled and double-blind crossover trial by Homan et al. showed that the relative increase in FT3 levels in patients with major depression was associated with reduced glucose metabolism in the right dorsolateral prefrontal cortex region [38]. A significant increase in thyroid stimulating hormone concentrations may be another risk factor for the development of type 2 diabetes in subjects with normal thyroid function [39]. Another study by Bos et al. showed that TSH, but not FT4, was a potential risk factor for DM in genetically determined individuals with low BMI [40]. Wu et al. reported that the HAMD score was one of the risk factors for underweight in Chinese newly diagnosed and drug-naïve patients with Parkinson’s disease [41]. Treatment in patients with co-morbid MDD and diabetes mellitus resulted in a decrease in HAMD score [42, 43].

In this study, we also found that TSH was independently associated with glucose concentration in MDD patients with FEDN with comorbid ATF, suggesting that the severity of thyroid function was an independent risk factor of higher glucose concentration in FEDN MDD patients with ATF. The reason for that may be there was a complex relationship between ATF and abnormal glucose and depression. SCH generally represents early mild thyroid failure and signals a risk of progression from subclinical hypothyroidism to overt hypothyroidism. Serum thyroid hormone levels are closely related to lipid metabolism, insulin metabolism, and inflammatory factors [44]. Therefore, we chose TSH to screen ATF in this study. Moreover, the association of co-morbidity ATF with abnormal glucose in never-treated MDD patients may be attributed to the altered HPT axis 40. HPT axis triggers autonomic dysfunction. Regardless of changes in thyroid hormone concentrations, fluctuations in blood glucose have a direct effect on TSH secretion [45]. But this is only our speculation and needs further verification.

Our study has some limitations. First, all patients were recruited from the psychiatric outpatient department of a general hospital in Taiyuan City. Therefore, our results should be more cautiously generalized to populations in other regions, races, or countries. Second, our study was a cross-sectional study, so we could not distinguish the causal relationship between abnormal glucose and MDD combined with ATF. Future multicenter prospective studies are needed. Third, in this study, we collected only fasting blood glucose levels, but did not perform an oral glucose tolerance test (OGTT), which is a common clinical criterion for diagnosing diabetes. Also, we did not collect the postprandial blood glucose indicators, which are also important for diagnosing impaired glucose metabolism. In future studies, OGTT should be conducted and the postprandial blood glucose indicators should be collected to remedy the limitation of this study. Fourth, in this study, according to our exclusion criteria, only patients with severe or unstable physical illnesses were excluded. For example, we excluded patients who had illnesses that seriously affected the patient’s physical and mental health, daily life, and daily communication. If a person suffered from diabetes or thyroid disorder, but has now recovered or in a stable state, he/she was not excluded from the study. However, in the current study, their history of physical illnesses had the potential to influence their glucose and thyroid levels in current study. Fortunately, our patients of first episode MDD patients were young, and we found that only a few patients had previous diabetes or thyroid disorder, which may not have significantly affected the results of this study. Fifth, it is known that many comorbid medical and mental illnesses may affect thyroid levels, such as Alzheimer’s Disease, brain injury, and unselected populations with acute episodes of psychosis [46–48]. However, not all of these diseases were specified in the exclusion criteria, which should be remedied in future studies. Fortunately, in this study, our patients were first-episode MDD patients who were relatively young, had a shorter disease duration, and had few comorbid medical and mental illnesses. Finally, we lacked a healthy control group in this study. It would be better to add a group of healthy controls, as we need to know the prevalence of ATF combined with abnormal glucose in healthy controls to further illustrate the prevalence and clinical profile of abnormal glucose in MDD with comorbid ATF. In future studies, we will add a group of healthy controls to compensate for the methodological limitation of this study.

## Conclusions

In conclusion, our study showed that the prevalence of abnormal glucose in FEDN MDD patients with comorbid ATF was 47.3%, indicating that abnormal glucose is very common in the early acute phase of Chinese MDD samples. Furthermore, our study showed that the prevalence of suicide attempts, severe anxiety, and psychotic symptoms was significantly higher in MDD patients with ATF who had abnormal glucose than those who had not abnormal glucose. In addition, higher HAMD scores and TSH levels were independently associated with abnormal glucose of MDD patients with comorbid ATF. Further, the main findings of this study have valuable clinical implications. First, in the prevention of abnormal glucose, MDD patients with comorbid ATF can be considered as a potential subtype of MDD. Specific treatment strategies should be developed for this potential subtype. Thus, the evaluation of blood glucose concentration in these patients should be made, if possible, by multiple samples on patients with MDD to avoid underestimation or overestimation of glucose metabolism disorders with consequent inappropriate choice of therapeutic options.

## Data Availability

The data that support the findings of this study are available from the corresponding author upon reasonable request.

## Abbreviations

MDD: Major depressive disorder
SCH: Subclinical hypothyroidism
FT3: Triiodothyronine
FT4: Free thyroxine
RMR: Resting metabolic rate
TSH: Thyroid-stimulating hormone
TGC: Tight glucose control
ATF: Abnormal thyroid function
FEDN: First-episode and drug-naïve
SCID: Structured Clinical Interview for DSM-IV
HAMD: Hamilton Rating Scale for Depression
IRB: Institutional Review Board
BMI: Body mass index
HAMA: Hamilton Anxiety Scale
TgAb: Anti-thyroglobulin
TPOAb: Thyroid peroxide antibody
ANOVA: Analysis of variance
AUC-ROC: Area under the receiver operating characteristic
